# Degeneracy and disordered brain networks in psychiatric patients using multivariate structural covariance analyzes

**DOI:** 10.3389/fpsyt.2023.1272933

**Published:** 2023-10-13

**Authors:** Rositsa Paunova, Cristina Ramponi, Sevdalina Kandilarova, Anna Todeva-Radneva, Adeliya Latypova, Drozdstoy Stoyanov, Ferath Kherif

**Affiliations:** ^1^Department of Psychiatry and Medical Psychology, Medical University Plovdiv, Plovdiv, Bulgaria; ^2^Research Institute, Medical University Plovdiv, Plovdiv, Bulgaria; ^3^Laboratory for Research in Neuroimaging, Department of Clinical Neuroscience, Lausanne University Hospital and University of Lausanne, Lausanne, Switzerland

**Keywords:** schizophrenia, major depressive disorder, bipolar disorder, neuroimaging, structural covariance

## Abstract

**Introduction:**

In this study, we applied multivariate methods to identify brain regions that have a critical role in shaping the connectivity patterns of networks associated with major psychiatric diagnoses, including schizophrenia (SCH), major depressive disorder (MDD) and bipolar disorder (BD) and healthy controls (HC). We used T1w images from 164 subjects: Schizophrenia (*n* = 17), bipolar disorder (*n* = 25), major depressive disorder (*n* = 68) and a healthy control group (*n* = 54).

**Methods:**

We extracted regions of interest (ROIs) using a method based on the SHOOT algorithm of the SPM12 toolbox. We then performed multivariate structural covariance between the groups. For the regions identified as significant in t term of their covariance value, we calculated their eigencentrality as a measure of the influence of brain regions within the network. We applied a significance threshold of p = 0.001. Finally, we performed a cluster analysis to determine groups of regions that had similar eigencentrality profiles in different pairwise comparison networks in the observed groups.

**Results:**

As a result, we obtained 4 clusters with different brain regions that were diagnosis-specific. Cluster 1 showed the strongest discriminative values between SCH and HC and SCH and BD. Cluster 2 had the strongest discriminative value for the MDD patients, cluster 3 – for the BD patients. Cluster 4 seemed to contribute almost equally to the discrimination between the four groups.

**Discussion:**

Our results suggest that we can use the multivariate structural covariance method to identify specific regions that have higher predictive value for specific psychiatric diagnoses. In our research, we have identified brain signatures that suggest that degeneracy shapes brain networks in different ways both within and across major psychiatric disorders.

## 1. Introduction

Over the past decades, a constant debate on the validity of psychiatric diagnosis exists (1, 2). Despite the development of advanced methods to explore brain structure and function, the translation of neuroimaging research into viable biomarkers of the psychiatric symptoms and syndromes, remains elusive (3). It became increasingly evident that we needed to move beyond simple voxel correlations to better relate our neuroscientific results to psychiatric nosologies and their symptoms. Consequently, contemporary theories of psychopathology recently focus on the disruption of neural circuits rather than on a particular brain area. There is increasing interest in exploring these network disruptions in the major psychiatric disorders such as schizophrenia (SCH), major depressive disorder (MDD), bipolar disorder (BD), etc. Identifying dysfunctions in particular brain networks may be the key to preventative and more precise treatments, and better outcome of various psychiatric conditions.

Network neuroscience is a rapidly expanding field (4, 5). One of the main conceptions comes from the application of mathematical and computational tools, developed for neurobiological systems, and the application of models that stem from graph theory (6, 7). Graph theory was established in the 18th century as a mathematical branch. Nowadays, it is widely used in a variety of scientific disciplines and computing technologies. By means of those methodologies and graph models, we are able to describe the properties of the structural and functional brain networks, a well as how these metrics are associated with different clinical representations (8, 9).

Structural covariance analysis based on structural magnetic resonance imaging (sMRI) within a graph-analysis approach that enables the identification of disruptions in different brain networks. The method has been used to explore brain morphology changes associated with various neurological and psychiatric diseases like Alzheimer’s, schizophrenia and depression (10, 11). Moreover, it is proposed that structural covariance reflects the coordination of maturation between areas of the brain which is known to be abnormal in major psychiatric disorders.

Schizophrenia is a disabling psychiatric condition characterized by dysconnectivity between and within different brain networks (12, 13). Moreover, it has been found that people suffering from SCH have various structural brain abnormalities including gray matter volume reductions (14, 15). Various evidence corroborates the presence of functional network disruptions, and additionally, structural integrity reduction in fronto-parietal control and salience networks has been reported in SCH (16). Lately, by means of structural covariance network (SCN) analysis, reduced integrity was found in insular-limbic, occipito-temporal, temporal, and parahippocampal-limbic networks (17).

Bipolar disorder is characterized by the presence of at least one manic (type I BD) or hypomanic episode (type II BD) most commonly appearing amongst recurrent depressive states. Brain structure is known to be affected in BD with core alterations encompassing dorsomedial and ventromedial prefrontal cortex, anterior cingulate cortex and bilateral insula (18). SCN research revealed recently that compared to MDD, BD demonstrated common decreases of the structural covariance (SC) between nucleus accumbens (Nac) connected to prefrontal gyrus, bilateral striatum, and anterior insula. However, they were characterized by distinct increases in SC of Nac connected to the left hippocampus and thalamus (19).

Both BD I and BD II had reduced SC connections between superior frontal gyrus (SFG), postcentral gyrus (PCG), superior temporal gyrus (STG), and pars opercularis, while only type I patients had decreased SC connections between STG, inferior parietal gyrus (IPG), pars opercularis (20). In contrast to SCH, BD patients showed increased clustering coefficients in the left suborbital sulcus and the right superior frontal sulcus (21).

Major depressive disorder is a condition also associated with disrupted brain networks not only for functional network but also, for structural covariance network. According to a most recent SCN study, hippocampus, thalamus and ventromedial prefrontal cortex (vmPFC) are reported to be the primary regions affected at the onset of illness with further influence on other brain regions including nucleus accumbens, the precuneus and the cerebellum on structural level (22). Interestingly, remitted psychotic depression has been linked to reductions mainly in cortico-limbic SCNs (23). Another study suggests that there is negative structural association between the left DLPFC and left amygdala and positive structural association between the bilateral DLPFC which was observed in controls and was absent in patients with MDD. This could lead to the conclusion that the discrepancies observed at the structural levels are connected, or lead to, dysfunctional brain network at the functional level (24).

To the best of our knowledge, at the time of the writing of the current text, there are no SCN studies directly comparing the three major psychiatric diagnosis – SCH, BD and MDD except for one ENIGMA consortium report based on cortical thickness (25). Aiming at filling this gap, we conducted a transdiagnostic SCN study on gray matter volume (GMV) measures of patients representing the above mentioned mental conditions and matched healthy controls. We hypothesized that there will be common as well as distinct SCN characteristics of the three patient groups. From the perspective of network neuroscience, structural covariance enables an assessment of degeneracy. Degeneracy is related to the degree of plasticity and resilience of the brain and is well suited to the case of brain diseases where symptoms may be common. The degeneracy principle, which states that different neural structures can produce similar functional outcomes, is particularly important when dealing with common symptoms that may have different neural bases in different diseases. In this study, degeneracy centrality and clustering methods are used to measure degeneracy and identify critical nodes based on similar connectivity patterns.

## 2. Materials and methods

### 2.1. Participants

For the study, 164 participants were included either with the following diagnosis – schizophrenia, major depressive disorder, bipolar disorder or as healthy volunteers. Each participant was assessed by experienced psychiatrist (D.S., S.K.) *via* a general clinical interview and the structured Mini International Neuropsychiatric Interview (MINI 6.0) (26) as well as the Montgomery–Åsberg Depression Rating Scale (MADRS) and the Positive and Negative Syndrome Scale (PANSS). Patients with depression with a total MADRS score of at least 20 were included, as well as psychotic patients with at least 3 on P1 (delusions) or P6 (suspiciousness) PANSS. Patients with bipolar disorder with mixed features were excluded. Both groups had been on stable medication for the past 14 days. We adhered to the following exclusion criteria: age under 18 years or over 65 years, presence of MRI-incompatible metal implants or body grafts (e.g., pacemaker), severe somatic or neurological disease, comorbid mental disorder (e.g., substance or alcohol use disorder, obsessive compulsive disorder, etc.), and traumatic brain injury with loss of consciousness. Each participant provided a written informed consent in compliance with the Declaration of Helsinki. The study protocol was approved by the University’s Ethics Committee.

### 2.2. Data

The scanning protocol was conducted on a 3T MRI system (GE Discovery 750w)using a: high-resolution structural scan (Sag 3D T1 FSPGR sequence), with a slice thickness of 1 mm, matrix 256 × 256, TR (relaxation time) of 7.2 ms, TE(echo time) of 2.3 ms, and flip angle 12°, This MRI sequence was utilized to estimate gray matter volume for the purpose of performing structural covariance analyzes. All brain scans were assessed by experienced radiologist, who excluded patients and healthy controls with signs of neurodegenerative diseases, malignant neoplasms, dementia, stroke, vascular diseases.

### 2.3. Methods

T1w images were preprocessed using the standard SPM12 pipeline for spatial bias correction and segmentation into gray matter, white matter, CSF, and other brain tissue priors. Gray matter images were parceled into multiple regions of interest (ROIs) based on the neuromorphometric atlas and using a parcellation method based on the SHOOT algorithm of the SPM12 toolbox. The volume of each brain region was extracted for each participant.

Multivariate Structural Analyzes of covariance between groups: Partial correlation matrices were created for each group. These matrices represented the structural covariance between the regions of interest (ROIs), taking into account age and gender. Each entry in the matrix embodied the unique relationship between a pair of ROIs, without the influence of these demographic factors. After creating the matrices, we conducted exhaustive pairwise comparisons between each disease group (SZ, MDD, BD) and the healthy control group (HC) and we also compared each disease group with each other. These statistical comparisons identify brain regions that showed significant differences in structural covariance between groups. For the regions identified as significant in these pairwise comparisons, we calculated their eigen centrality to quantify the influence of each node (brain region) within the network. Finally, we performed a cluster analysis method to identify groups of regions that had similar eigen-centrality profiles in different pairwise comparison networks. For the clustering analysis, we first constructed a matrix in which each node was represented as a vector of its eigen centrality values across the six pairwise comparison networks. Each row of this matrix corresponded to a node and each column represented one of the six networks. We applied k-means clustering to the matrix to categorize the nodes into distinct clusters. The optimal number of clusters, k, was determined by the Elbow Method, based on explained variance against the number of clusters.

## 3. Results

### 3.1. Demographic and clinical characteristics

The demographic and the clinical characteristics of the 164 subjects with schizophrenia (*n* = 17), bipolar disorder (*n* = 25), major depressive disorder (n = 68) and a healthy control group (*n* = 54) are reported in Table 1. The clinical characteristics from the assessments scales are as follows: MADRS score (mean ± SD) for the Healthy control group (HC) is 1.6 ± 2.1; in the context of bipolar disorder (BD) is 28.1 ± 5.0; in the context of major depressive disorder (MDD) is 30 ± 6.5., PANSS score for the patients with Schizophrenia (SCH) is 57 ± 13.6. There were no significant differences in age, sex, education level between HC, SCH, BD and MDD groups.

**Table 1:** Demographic and clinical characteristics of the samples.

	Healthy controls (*n* = 54)	Bipolar patients (*n* = 25)	MDD patients (*n* = 68)	Schizophrenia patients (*n* = 17)	*p*-value
Age (mean, SD)	39.6 ± 12.4	41.1 ± 9.6	44.2 ± 13.9	36.7 ± 12.2	0.06^a^
Sex (M/F)	17/37	6/16	16/52	9/8	0.11^b^
Education (years)	15.1 ± 3.2	14.08 ± 2.2	14.2 ± 2.5	13.1 ± 2	0.056^b^[L1]

SD, standard deviation. ^a^ Independent samples *t*-test, ^b^ *χ*^2^ test.

### 3.2. Multivariate graph method results

**Figure 1:**
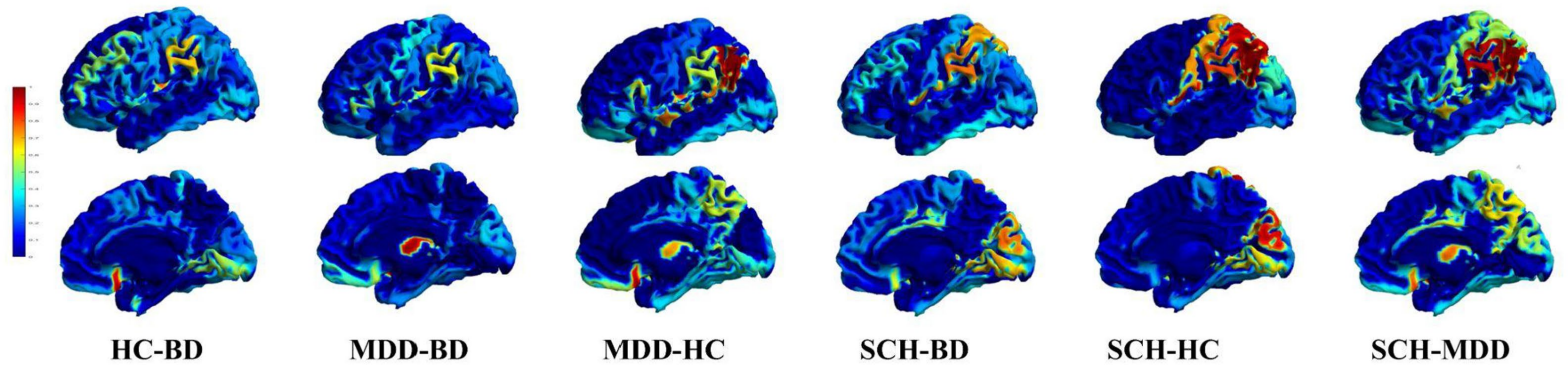
Critical nodes and their relative influence within and between different disease comparisons. 3D mesh projections displaying node eigen centrality different pairwise comparisons between disease groups. Nodes are color-coded based on their eigencentrality values. The top row shows the left view, while the bottom row shows the lateral view. From left to right, the columns illustrate pairwise comparisons for HC-BD, MDD-BD, MDD-HC, SCH-BD, SCH-HC and SCH-MDD. This visualization highlights critical nodes and their relative influence within and between different disease comparisons.

Following the calculation of the structural covariances matrices within each group and the pairwise comparisons, the alpha level was set at 0.001. This threshold was determined using a permutation test to compare the correlation matrices. Consequently, 61 regions were identified. In Table 2, we report the eigen-centrality for each of these significant regions within each pairwise comparison network. The brain projections of the eigen centrality are presented in Figure 1.

**Table 2:** The table presents the eigen centrality of the 61 identified regions which had statistically significant differences between the groups.

Cluster ID	Region name	Average	HC-BD	MDD-BD	MDD-HC	SCH-BD	SCH-HC	SCH-MDD
1	SMG supramarginal	0.0386	0.0366	0.0375	0.0274	0.0413	0.0468	0.0421
	AOrG ant orbital	0.0333	0.0164	0.0164	0.0476	0.0164	0.0578	0.0451
	PO parietal operculum	0.0294	0.0214	0.0050	0.0233	0.0445	0.0478	0.0343
	Cun cuneus	0.0258	0.0128	0.0208	0.0004	0.0422	0.0528	0.0255
	Hippocampus	0.0251	0.0210	0.0097	0.0004	0.0543	0.0440	0.0212
	SPL sup parietal	0.0239	0.0139	0.0083	0.0049	0.0386	0.0528	0.0245
	Calc calcarine	0.0224	0.0123	0.0058	0.0005	0.0395	0.0528	0.0232
	LOrG lateral orbital	0.0218	0.0302	0.0010	0.0211	0.0382	0.0374	0.0028
	PrG precentral	0.0210	0.0164	0.0164	0.0112	0.0164	0.0416	0.0243
2	PoG postcentral	0.0400	0.0154	0.0614	0.0361	0.0405	0.0416	0.0451
	SCA subcallosal area	0.0379	0.0543	0.0346	0.0480	0.0343	0.0182	0.0380
	PHG parahippocampal	0.0291	0.0431	0.0405	0.0404	0.0092	0.0149	0.0263
	CO central operculum	0.0276	0.0255	0.0319	0.0324	0.0252	0.0164	0.0345
	TrIFG triangular inf front	0.0229	0.0006	0.0589	0.0345	0.0038	0.0048	0.0348
3	MFG mid front	0.0128	0.0288	0.0116	0.0036	0.0220	0.0026	0.0080
	MPoG postcentral medial	0.0126	0.0221	0.0204	0.0042	0.0175	0.0070	0.0047
	POrG post orbital	0.0113	0.0164	0.0164	0.0164	0.0069	0.0058	0.0061
	FO front operculum	0.0113	0.0164	0.0174	0.0164	0.0018	0.0029	0.0131
	MFC medial front	0.0111	0.0009	0.0292	0.0121	0.0118	0.0067	0.0061
	Pallidum	0.0105	0.0164	0.0164	0.0164	0.0050	0.0048	0.0038
	PT planum temporale	0.0104	0.0164	0.0241	0.0089	0.0037	0.0001	0.0091
	AnG angular	0.0088	0.0095	0.0060	0.0164	0.0038	0.0007	0.0164
	Caudate	0.0086	0.0164	0.0164	0.0016	0.0164	0.0002	0.0004
	OrIFG orbital inf front	0.0085	0.0079	0.0132	0.0164	0.0081	0.0051	0.0003
	PIns post insula	0.0083	0.0086	0.0051	0.0164	0.0170	0.0021	0.0004
	AIns ant insula	0.0081	0.0164	0.0016	0.0085	0.0045	0.0164	0.0010
	PCu precuneus	0.0078	0.0024	0.0121	0.0164	0.0049	0.0074	0.0036
	MSFG sup front medial	0.0070	0.0076	0.0081	0.0004	0.0153	0.0061	0.0043
	SMC supp motor	0.0064	0.0155	0.0088	0.0000	0.0104	0.0013	0.0021
	Thalamus Proper	0.0057	0.0041	0.0144	0.0026	0.0036	0.0056	0.0041
	SFG sup front	0.0056	0.0045	0.0058	0.0010	0.0141	0.0026	0.0054
	MTG mid temporal	0.0048	0.0041	0.0064	0.0024	0.0053	0.0035	0.0071
4	TMP temporal pole	0.0223	0.0164	0.0164	0.0365	0.0164	0.0172	0.0310
	STG sup temporal	0.0198	0.0164	0.0164	0.0370	0.0164	0.0021	0.0305
	PP planum polare	0.0190	0.0164	0.0164	0.0319	0.0164	0.0164	0.0164
	OpIFG opercular inf front	0.0182	0.0175	0.0185	0.0290	0.0141	0.0108	0.0193
	FRP front pole	0.0167	0.0164	0.0164	0.0184	0.0164	0.0164	0.0164
	MOrG medial orbital	0.0165	0.0164	0.0164	0.0171	0.0164	0.0164	0.0164
	Basal Forebrain	0.0164	0.0164	0.0164	0.0164	0.0164	0.0164	0.0164
	CerebVermal I0x2DV	0.0164	0.0164	0.0164	0.0164	0.0164	0.0164	0.0164
	Cerebellum Exterior	0.0164	0.0164	0.0164	0.0164	0.0164	0.0164	0.0164
	OCP occipital pole	0.0164	0.0164	0.0164	0.0164	0.0164	0.0164	0.0164
	IOG inf occipital	0.0158	0.0187	0.0082	0.0164	0.0185	0.0164	0.0164
	Ent entorhinal area	0.0157	0.0302	0.0164	0.0171	0.0086	0.0182	0.0034
	MCgG mid cingulate	0.0153	0.0158	0.0032	0.0154	0.0304	0.0000	0.0271
	GRe rectus	0.0153	0.0164	0.0275	0.0261	0.0035	0.0001	0.0182
	SOG sup occipital	0.0146	0.0164	0.0164	0.0031	0.0164	0.0245	0.0107
	Amygdala	0.0144	0.0349	0.0144	0.0121	0.0055	0.0168	0.0027
	FuG fusiform	0.0143	0.0041	0.0164	0.0164	0.0164	0.0164	0.0164
	TTG transverse temporal	0.0143	0.0164	0.0332	0.0154	0.0004	0.0008	0.0197
	Cereb Vermal VIII DX	0.0138	0.0164	0.0164	0.0164	0.0164	0.0164	0.0010
	Vermal VI0 DVII	0.0138	0.0164	0.0164	0.0164	0.0008	0.0164	0.0164
	Accumbens Area	0.0137	0.0164	0.0164	0.0000	0.0164	0.0164	0.0164
	ITG inf temporal	0.0136	0.0156	0.0104	0.0164	0.0211	0.0019	0.0164
	LiG lingual	0.0136	0.0164	0.0164	0.0164	0.0140	0.0019	0.0164
	ACgG ant cingulate	0.0134	0.0239	0.0028	0.0164	0.0047	0.0164	0.0164
	MOG mid occipital	0.0132	0.0101	0.0179	0.0031	0.0119	0.0257	0.0108
	Putamen	0.0129	0.0101	0.0164	0.0085	0.0086	0.0176	0.0164
	uG occipital fusiform	0.0125	0.0164	0.0024	0.0164	0.0070	0.0164	0.0164
	PCgG post cingulate	0.0117	0.0018	0.0012	0.0277	0.0048	0.0056	0.0289
	MPrG precentral medial	0.0116	0.0041	0.0002	0.0164	0.0164	0.0164	0.0164

**Figure 2:**
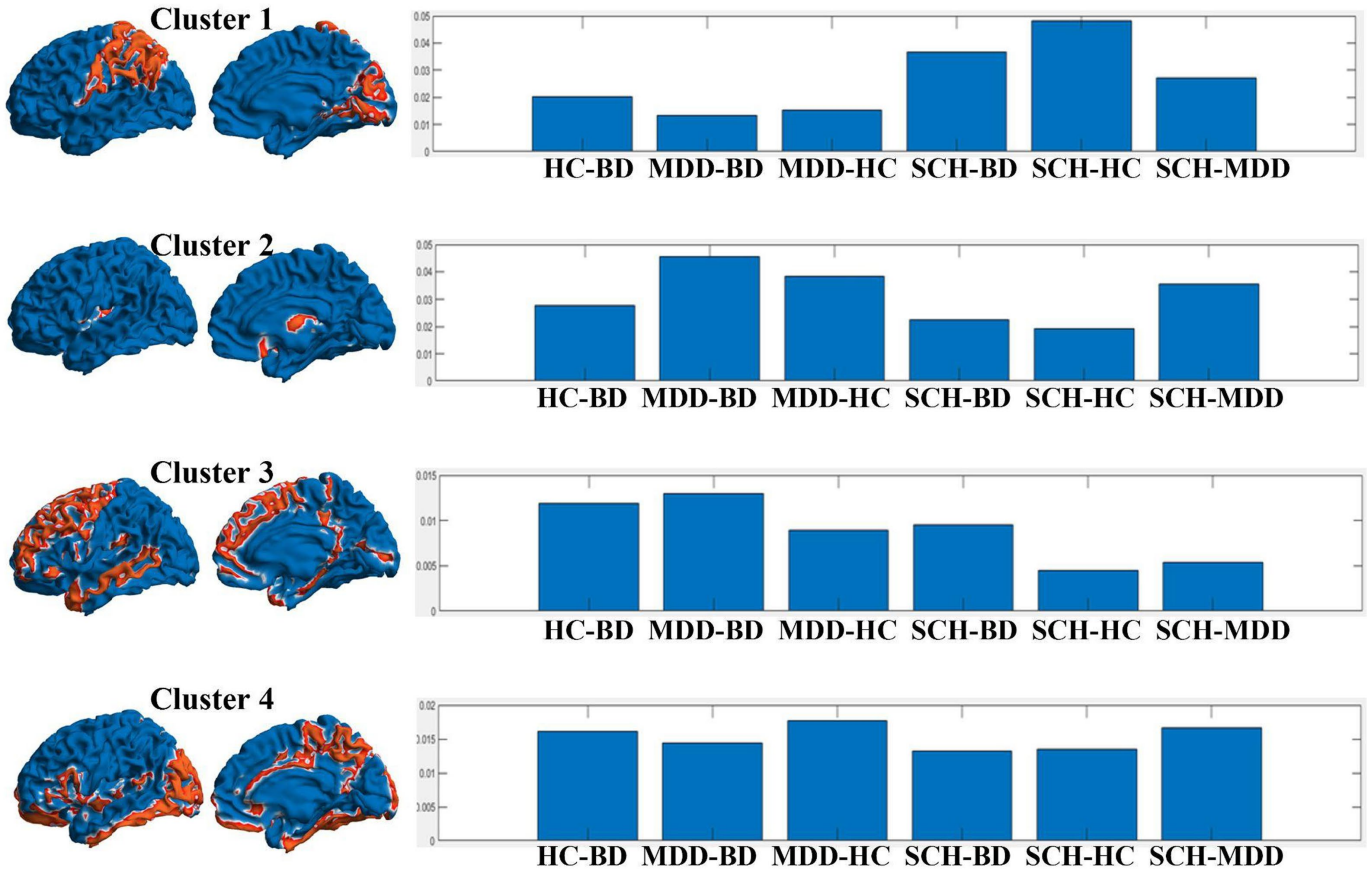
Representation of the clusters derived from the analysis, juxtaposed with their eigen centrality profiles in different pairwise comparisons between disease groups. The left panel of each row shows two 3D mesh views (left view and inner view) of the brain, with the regions belonging to each cluster highlighted in red. The right panel of each row shows the eigen centrality profile of the corresponding cluster across the pairwise comparisons: HC-BD, MDD-BD, MDD-HC, SCH-BD, SCH-HC, and SCH-MDD.

For the clustering analysis, we identified the optimal number of clusters (k) using the Elbow Method, which was found at k = 4. By adopting k = 4 for our clustering analysis, we were able to delineate four distinct clusters of regions, also shown in Table 2; Figure 2 (The rows show clusters 1 to 4 in succession, allowing a comparative visualization of the spatial distributions and associated eigen centrality profile).

For example, the profile of the first cluster appeared show higher values for SCH-HC, as well as SCH-BD, and with smaller values for SCH-MDD contrast. The cluster encompasses brain areas belonging to parietal (operculum, supramarginal gyrus – SMG, superior parietal lobule – SPL), occipital (cuneus, calcarine cortex), frontal (precental, anterior and lateral orbital gyri), and temporal lobes (hippocampus).

The regions from the second cluster have the higher value for the difference MDD- vs. all others groups and encompass postcentral gyrus, subcallosal area, parahippocampal gyrus, central operculum and the triangular part of the inferior frontal gyrus.

The third cluster was contributing most for the discrimination of the BD patients with distributed regions within frontal (superior, middle and inferior frontal gyrus – pars orbitalis, supplementary motor area, operculum, medial frontal cortex, posterior orbital gyrus), parietal (medial segment of the postcentral gyrus, angular gyrus, precuneus), temporal lobes (anterior and posterior insula, planum temporale, middle temporal gyrus), and subcortical structures such as pallidum, caudate and thalamus.

Last, the regions within the fourth cluster seem to contribute almost equally to the differentiation between the four groups. Here the brain areas involved are numerous and span across structures such as amygdala, nucleus accumbens, putamen, basal forebrain, anterior, middle and posterior cingulate cortex, entorhinal area, frontal, temporal and occipital pole, planum polare, cerebellum, middle and inferior temporal gyri, superior, middle and inferior occipital gyri, fusiform and lingual gyri.

## 4. Discussion

The results of this study based on structural covariance and cluster analysis in SCZ, BD, MDD and HC revealed four significantly different clusters of brain areas ranked on their “authorities,” “hubs” and “eigen mean” measures. The first cluster contributed most to the differentiation of SCZ patients from HC, BD and MDD, while the second cluster was more relevant to the MDD group, and the third cluster – to the BD sample. The fourth cluster represented regions which were shared across diagnostic groups. In this particular study our idea was to investigate the depressive state and the changes in brain networks that it leads to. On account of that we decided not to focused on the affective disorder spectrum, respectfully we did not include bipolar patients with manic episodes. The significance of these findings will be discussed in the following lines.

Cluster 1 has a prominent contribution for discriminating of schizophrenia from both bipolar disorder, healthy controls, and to a slightly lesser degree from MDD patients. These results may be interpreted in terms of sensitivity (discrimination of health from disease) and specificity (discrimination of two different disease populations), e.g., the regions within this cluster demonstrate both. These are namely: SMG, SPL, cuneus, calcarine cortex, precentral gyrus, anterior and lateral orbital gyri, and hippocampus. Being involved in various functions including somatosensory association, language, empathy, visual processing, working memory, attention, emotion, episodic and spatial memory, these brain areas seem to be differentially affected in SCZ as compared to the other groups in our study. In other words, the gray matter volume changes in these regions are correlated in such a way that discriminates this major psychotic disorder from both mood disorders and healthy individuals.

Our findings are in line with various research demonstrating the involvement of the abovementioned brain structures in the pathophysiology of SCZ. The parietal operculum is considered a hub, in which several sensory-motor streams originating from different cerebral areas converge, and it is a part of the cingulo-opercular network (CON) along with anterior insula, other divisions of operculum, dorsal anterior cingulate cortex, and thalamus (27). CON is thought to facilitate the maintenance of task-relevant goals and the incorporation of error information to adjust behavior (28) and it has been found to be dysfunctional in SCZ (29). Moreover, gray matter reduction of parietal operculum has been linked to treatment resistance in SCZ (30).

SMG is part of the inferior parietal lobule (IPL) which is known to be amongst the last brain areas to mature, e.g., to myelinate (31). Amongst other regions, reduction of GM volume of SMG has been found in first episode SCZ as compared to both healthy controls and genetic high-risk individuals (32). Moreover, GM density as well as gyrification, cortical surface area and thickness were reported to be decreased in schizophrenic patients in compared to healthy subjects (33). Interestingly, the gyrification index correlated negatively with the disorganization score of the patients while SMG thickness was negatively related to illness duration (34). Thus, SMG GM alterations seem to be a feature of the development and progression of schizophrenic psychosis.

The SPL, on the other hand, plays an important role in different brain functions including visuomotor, cognitive, sensorimotor integration, working memory and attention (35). Based on extensive research demonstrating various structural and functional disturbances of the parietal lobes in non-affected siblings and ultrahigh risk individuals, as well as in SCZ patients, it was proposed that in some cases, (especially in early onset) SCZ is accompanied by, structural and functional alterations starting in the parietal lobes, and progressing to the frontal regions (36).

The other significant region in cluster 1 is the cuneus where primary visual processing occurs. Interestingly, this area has been found to be increased in a number of voxel-based morphometry studies of psychosis onset and progression (37, 38). A Possible explanation of this finding might be the compensatory activation of the visual cortex that has been found in patients during working memory tasks (39). This aberrant activation was associated with better working memory capacity. Recent meta-analysis of emotion perception studies demonstrated consistent visual cortex hyperactivation in SCZ (40).

The OFC has been implicated in various multimodal functions spanning across sensory-visceromotor integration, affective evaluation of rewards and punishments, expectation, motivation, decision-making, goal-directed and social behavior (41). SCZ has been linked to structural alteration of OFC including reduced GM volume and thickness, as well as white matter abnormalities (42–45). Left medial OFC thickness was significantly associated with negative symptom severity in a recent meta-analysis by the ENIGMA consortium (46).

The last region in Cluster 1 is the hippocampus known for its crucial role in memory and emotion, with clear implications in the pathogenesis of schizophrenia, relevant to the degenerative nature and progressive course of the disease (47). Smaller volume of the hippocampal formation has been consistently reported in SCZ and evidence exists for a link between the structural alterations and the cognitive dysfunction, especially impaired episodic memory (48, 49). In sum, all cluster 1 regions have been found to demonstrate structural alterations in SCZ, mostly GM volume reductions except for the cuneus where an increase has been reported. In addition, the volume changes have been linked to the severity of the symptoms and to the progression of the illness. Other structural covariance studies have found similar results regarding for example reduced covariance in the orbitofrontal cortex area in early psychosis compared to HC (50). Zhang et al. (51) reported differences in the slope of structural covariance networks between SZ and HC within the auditory and executive control networks. They found auditory regions to be positively correlated in controls but negatively or not correlated in patients, while the executive control network had an opposite pattern, with positive correlations in SCZ and negative or no correlations in HC. Other researchers found reductions in structural integrity of the fronto-parietal control and salience networks (52).

The regions that belong to Cluster 2 had the most outstanding contribution to the diagnosis of MDD. Notably they include the parahippocampal gyrus, triangular part of inferior frontal gyrus (IFG), the subcallosal area, and the central operculum. Those areas underpin mental functions related to memory, language processing and understanding of the social context, which are both impaired in depression at the functional as well as at the structural level (53, 54). For example, the gray-matter volume (GMV) reduction of the parahippocampus has been correlated with the severity of depressive symptoms in Alzheimer’s disease (55). In addition, the activity of the parahippocampus has been associated with a weaker response to reward stimuli in patients with MDD as opposed to HC (56). Another study suggests that the increased activity in the parahippocampal area could predict the response to treatment with SSRIs (57). The aforementioned research is in line with our results suggesting that the structural alterations of the parahippocampus may not only underlie some of the functional changes observed in MDD but may also have a discriminative diagnostic value for this particular disorder.

The other significant region in cluster 2 was the triangular part of the inferior frontal gyrus. Volumetric changes as well as altered connectivity and activity patterns in the inferior frontal gyrus have been implicated in the pathophysiology of MDD. In adolescents with depression for example, an increase in the gray-matter volume was observed in the IFG along with the cingulate gyrus, thalamus, superior frontal gyrus, middle frontal gyrus, and the superior and inferior temporal gyri as opposed to HC (58). Moreover, a systematic review showed that altered activity of the IFG and the dorsolateral prefrontal cortex during emotional tasks may be depression-specific (59). Another study demonstrated a common aberration in patients with MDD and patients with mild cognitive impairment, namely decreased gray-matter volume of the IFG, insula, superior temporal gyrus, amygdala, thalamus, and hippocampus as compared to healthy individuals (60). Considering the substantial role of the IFG in basic cognitive functions such as language processing and the associated alterations of this structure with regions linked with executive functions, we may suggest the possibility that these changes are an underlying mechanism for the transient cognitive deficits observed in MDD. However, the existing volumetric alterations reported in literature may suggest a more complex role of this structure in terms of the phenomenology of depression that extends beyond the cognitive impairment towards domains such as cognitive appraisal of perceived language, social cues, etc. In addition, the novel drug intervention, ketamine has been recently demonstrated to normalize the structural changes induced in IFG by depression (61), which denotes future possibility of establishing this region as a therapeutic target in MDD.

A prominent finding in our research is the involvement of the anterior cingulate cortex (ACC) and more specifically the subcallosal area as well as the postcentral gyrus as differentiating components for MDD. The ACC has an extensive functional capacity with processing the affective component of pain (62) which is one of the more relevant in terms of the clinical presentation of depression. The postcentral gyrus, on the other hand, has been linked with the sensory-discriminative component of pain. In a previous study our research group found increased resting-state functional connectivity between the ACC and the postcentral gyrus in patients with depression in the context of both MDD and BD as opposed to healthy controls and we hypothesized that this finding may be interpreted as an impairment reflecting the signature of mental pain in depression (63). Moreover, a reduction of the gray-matter volume of the subgenual ACC was demonstrated in MDD but not in BD and healthy individuals which further supports the significance of this region for the etiopathophysiology of MDD (64).

Cluster 3 regions are characterized by greater contribution to the diagnosis of BD. They seem to form a distributed network of structural alterations in several frontal (superior - SFG, middle – MFG and IFG, supplementary motor area – SMA, operculum, medial frontal cortex, posterior orbital gyrus), parietal (medial segment of the postcentral gyrus, angular gyrus, precuneus), and temporal areas (anterior and posterior insula, planum temporale, middle temporal gyrus), along with subcortical structures (pallidum, caudate and thalamus).

Interestingly, our results reveal a significant involvement of regions from the Default Mode Network (DMN), the frontoparietal, salience, and limbic networks as discriminative components for BD, which is concordant with the literature. For example, a meta-analysis by Long et al. (65) demonstrated GMV changes in the default mode and cortico-striato-cerebellar network and more specifically increased GMV in the posterior cingulate cortex, striatum, and cerebellum as well as reduced GMV of the medial frontal gyrus and gyrus rectus in patients with first-episode BD compared to healthy controls (65). Another more recent meta-analytic data confirms decreased GM volumes in the right IFG extending to the right insula, temporal pole and superior temporal gyrus, left superior temporal gyrus extending to the left insula, temporal pole, and IFG, anterior cingulate cortex, left superior frontal gyrus (medial prefrontal cortex), left thalamus, and right fusiform gyrus (66). On the other hand, a structural covariance analysis in adolescent patients with BD type I and type II showed decreased connections between the SFG and the right postcentral gyrus, between the left superior temporal gyrus (STG) and the right poscentral gyrus, and between the left STG and right pars opercularis in comparison to healthy individuals. The differential modulation of the core symptom anhedonia by structural covariance networks of the nucleus accumbence was found in a comparison between patients with BD and MDD in a depressive episode. In particular, the severity of anhedonia was associated with the structural covariance network of the nucleus accumbence and amygdala, anterior insula, anterior cingulate cortex, and the caudate in MDD, whereas in BD the alterations were mainly in the prefrontal cortex and the striatum. Therefore, the evidence suggests that there is an involvement of at least three major networks in the pathophysiology of BD.

The heretofore discussed findings imply that there are both common and distinct patterns of structural and functional aberrations in schizophrenia, major depressive disorder, and bipolar disorder. These changes may not only explain some of the symptomatological characteristics of these disorders but may also inform future research directions in the search for diagnostic and therapeutic targets for mental illness. For instance, the dys-connectivity of the middle frontal gyrus, precuneus and angular gyrus are consistent with the findings from our earlier studies on the diagnosis of psychosis (67, 68). Structural alterations in the same areas have also been reported in patients with bipolar disorder as compared with healthy controls at baseline (69).

In cluster 4, the regions present the global picture of disrupted networks regardless of the diagnosis. We hypothesize that those regions that are less specific for our nosological classes might be involved in the overall degenerative course of mental disorders. According to the unitary psychosis concept (70, 71) there exist a continuum, or super-spectrum of psychosis (the spectrum of affective disorders and the schizophrenia spectrum). The general vector of this continuum is degeneracy in the common sense, i.e., progression of the deficit in terms of neurodegenerative alteration of the brain and corresponding cognitive and social-affective deficits. From that perspective the co-variant alterations in regions of cluster 4 are likely to represent the basic morphological substrate of disease.

Our analysis was based on the extraction of eigenvalues and egeinvectors from the voxels of the regions. Unlike volumetric measurements of individual voxels or the average, which are more sensitive to small variations, this method captures the significance of specific patterns within the data. This can provide some robustness to small volumetric perturbations associated with medication use. As most patients receive pharmacological treatment over a long period of time and take a wide range of medications, it is difficult to fully account for the perturbations that may result from taking these medications. Because of the large number of types of medications and the heterogeneity of their use, it is also difficult to use them as covariates in statistical models, as their estimates will be imprecise. We believe that the method we have chosen offers a degree of robustness to such effects, but we recognize the importance of taking this limitation into account in our discussion. By using multivariate methods, eigenvalues and interindividual differences in a few principal components can be calculated. In the future, when a large sample is analyzed, these components can be systematically compared with medication types to see if they influence the results.

The concept of degeneracy has emerged as an important perspective in neuroscience. According to this concept, different neural pathways or elements can lead to similar functional outcomes, providing the brain with a high degree of flexibility and resilience. In this study, eignecentrality and clustering methods are utilized to further understand these complex processes that are closely related to degeneracy. Based on similar patterns of connectivity across diseases, clusters of influential nodes can be seen to be a reflection of the robustness of the brain and its adaptability. In the future, extension of these methodological approaches will likely reveal the mechanisms related to the maintenance functionality despite potential perturbations, which is particularly significant for understanding psychiatric disorders.

## 5. Conclusion

Overall structural hyperconnectivity was observed in healthy controls and hypoconnectivity in patients with bipolar disorder and schizophrenia. This outlines a clear trajectory towards the unitary psychosis spectrum in terms of degeneracy, with the major depressive disorder identified closer to the healthy population whereas schizophrenia and BD outlining the spectrum of psychosis with various levels of disturbed structural connections. The alterations of structural connectivity have been regarded as crucial for understanding of the mechanisms behind schizophrenia (72, 73) but have been so far investigated mainly at the level white matter anomalies, i.e., fiber tracts imaging (DTI).

## 6. Limitations

One limitation of this study to be acknowledged is the relatively imbalanced sample, where fewer patients with schizophrenia were included in it. However it meets the criteria for statistical significance which is employed in most of the current neuropsychiatric imaging literature.

The second limitation is entailed by the pro-innovative methodology of the study, which is difficult to compare critically against earlier contributions in the field.

The third limitation is that most of the patients received long-term medication, which may be a confounding factor. However, the pharmacological treatment of this large scale of mental disorders is too heterogeneous (including antipsychotics, mood stabilizers, antidepressants from various generations) to be entered as covariate in the analysis (74, 75), as discussed earlier.

## Data availability statement

The original contributions presented in the study are included in the article/supplementary material, further inquiries can be directed to the corresponding author.

## Ethics statement

The studies involving humans were approved by Medical University of Plovdiv Ethics Committee on 29 May 2015 (ID: P-369/29.05.2015). The studies were conducted in accordance with the local legislation and institutional requirements. The participants provided their written informed consent to participate in this study.

## Author contributions

RP: Conceptualization, Data curation, Formal analysis, Methodology, Writing – original draft. CR: Methodology, Software, Validation, Writing – review & editing. SK: Supervision, Validation, Writing – original draft, Writing – review & editing. AT-R: Investigation, Supervision, Validation, Writing – original draft. AL: Methodology, Software, Validation, Writing – review & editing. DS: Supervision, Validation, Writing – review & editing. FK: Conceptualization, Methodology, Supervision, Validation, Writing – original draft.
